# *Arabidopsis ALA1* and *ALA2* Mediate RNAi-Based Antiviral Immunity

**DOI:** 10.3389/fpls.2017.00422

**Published:** 2017-04-07

**Authors:** Biyun Zhu, Hua Gao, Gang Xu, Dewei Wu, Susheng Song, Hongshan Jiang, Shuifang Zhu, Tiancong Qi, Daoxin Xie

**Affiliations:** ^1^Tsinghua-Peking Joint Center for Life Sciences, and MOE Key Laboratory of Bioinformatics, School of Life Sciences, Tsinghua UniversityBeijing, China; ^2^Beijing Key Laboratory of Plant Gene Resources and Biotechnology for Carbon Reduction and Environmental Improvement, College of Life Sciences, Capital Normal UniversityBeijing, China; ^3^The Institute of Plant Quarantine, Chinese Academy of Inspection and QuarantineBeijing, China

**Keywords:** ALA, *Arabidopsis*, 2b, CMV, RNA interference (RNAi), virus

## Abstract

RNA intereferencing (RNAi) pathway regulates antiviral immunity and mediates plant growth and development. Despite considerable research efforts, a few components in RNAi pathway have been revealed, including ARGONAUTEs (AGOs), DICER-LIKEs (DCLs), RNA-dependent RNA polymerase 1 and 6 (RDR1/6), and ALTERED MERISTEM PROGRAM 1 (AMP1). In this study, we performed a forward genetic screening for enhancers of *rdr6* via inoculation of CMV2aTΔ2b, a 2b-deficient Cucumber Mosaic Virus that is unable to suppress RNAi-mediated antiviral immunity. We uncover that the membrane-localized flippase Aminophospholipid ATPase 1 (ALA1) cooperates with RDR6 and RDR1 to promote antiviral immunity and regulate fertility in *Arabidopsis*. Moreover, we find that ALA2, a homolog of ALA1, also participates in antiviral immunity. Our findings suggest that ALA1 and ALA2 act as novel components in the RNAi pathway and function additively with RDR1 and RDR6 to mediate RNAi-based antiviral immunity and plant development.

## Introduction

RNA interference (RNAi) mediates plant defense against virus infections (Ding et al., 2004; Incarbone and Dunoyer, 2013; Martinez de Alba et al., 2013). DICER-LIKE ribonucleases (DCLs), such as DCL4, generate viral short interferencing RNAs (siRNAs) (Blevins et al., 2006; Parent et al., 2015), which direct the loading of viral RNAs into ARGONAUTE (AGO) proteins (e.g., AGO1) of the RNA-induced silencing complex (RISC) for the cleavage of viral RNAs (Morel, 2002; Adenot et al., 2006; Arribas-Hernandez et al., 2016), resulting in RNAi-mediated antiviral immunity. RNA-dependent RNA polymerases (RDRs) (Xie et al., 2001; Talmor-Neiman et al., 2006; Cao et al., 2014), including RDR1 and RDR6, promote synthesis of siRNAs by synthesizing long double-strand RNAs (dsRNAs), contributing to the antiviral immunity (Qu et al., 2008; Garcia-Ruiz et al., 2010).

Viruses in turn evolve viral suppressor of RNAi (VSR) to suppress host antiviral immunity. For example, Cucumber Mosaic Virus (CMV) utilizes the VSR protein 2b (Zhang et al., 2006; Diaz-Pendon et al., 2007) to suppress host RNAi-based antiviral immunity and causes severe pathogenic responses in wild-type *Arabidopsis*, while CMV2aTΔ2b, a CMV mutant without expression of 2b protein, is unable to cause any obvious viral symptoms in wild-type and the single mutants of *RDR1* or *RDR6*, but is able to cause severe pathogenic responses in the RNAi-deficient double mutant *rdr1 rdr6* (Wang et al., 2010).

Aminophospholipid transporting ATPases (ALAs) are membrane-localized flippases that are responsible for transporting different lipids, which is essential for asymmetry of membrane lipid bilayers (Lopez-Marques et al., 2010, 2012; Botella et al., 2016). There are 12 *Arabidopsis thaliana* ALAs in the IV subfamily of ATPases that control plant development or tolerance to temperature stresses (Lopez-Marques et al., 2014). ALA1 is required for plant tolerance to chilling (Gomes et al., 2000). ALA3 regulates pollen germination and pollen tube growth, and adaptability to chilling (Poulsen et al., 2008; McDowell et al., 2013). ALA6 and ALA7 control temperate-regulated pollen tube elongation (McDowell et al., 2015). ALA10 affects lipid uptake to regulate root growth and stomatal control (Botella et al., 2016).

In this study, we performed a forward genetic screening for enhancers of *rdr6* with CMV2aTΔ2b infection on M2 population of ethyl methanesulfonate (EMS)-mutagenized *rdr6*. We show that ALA1 and ALA2 act additively with RDR1 and RDR6 to mediate RNAi-mediated antiviral immunity and development. Our findings discover novel roles of ALA1 and ALA2.

## Materials and Methods

### Materials and Growth Conditions

The *Arabidopsis thaliana* mutants *ala1-2* (Salk_056947), *ala3* (GK-317H04), *ala7* (Salk_125598) and *ala10* (Salk_024877) were obtained from Arabidopsis Biological Resource Center. The *Arabidopsis* mutants *rdr1-1* (SAIL_672_F11), *rdr6-15* (SAIL_617H07), *rdr1 rdr6*, the L1 line transgenic for GUS, and the 2b-deficient CMV mutant CMV2aTΔ2b were described as previously (Boutet et al., 2003; Wang et al., 2010). The *ala1-2 rdr1, ala1-2 rdr6* and *ala1-2 rdr1 rdr6* were generated via genetic crossing. *Nicotiana benthamiana* was grown under a 16-h (28°C)/8-h (22°C) light/dark condition.

For observation of growth defects in **Figure 5C** and fertility and siliques development in **Figure 3**, *Arabidopsis* seeds were sterilized with 20% bleach, plated on Murashige and Skoog (MS) medium, chilled at 4°C for 3 days, and transferred to a growth room under a 16-h (23–25°C)/8-h (18–20°C) light/dark photoperiod for 9 days. The 9-day-old seedlings were transplanted into soil and grew in the same growth room for another ∼3 or ∼6 weeks.

### Viral Infection

For viral infection assays, *Arabidopsis* seedlings were sterilized, plated on MS medium, chilled at 4°C for 3 days, and transferred to a growth room under a 16-h (23–25°C)/8-h (18–20°C) light/dark photoperiod for 9 days. The 9-day-old seedlings were transplanted into soil for growth of another 14 days in a growth room under an 8-h (22–24°C)/16-h (16–19°C) light/dark photoperiod. The 23-day-old plants were inoculated with CMV2aTΔ2b as described previously (Wang et al., 2010), and the disease symptoms were recorded at 21 or 45 days after infection.

### EMS Mutagenesis

About 20,000 seeds (M1) of the *Arabidopsis* mutant *rdr6-15* (SAIL_617H07) were soaked with 100 mM phosphate buffer (pH 7.5) overnight at 4°C, washed with sterilized water for five times, and mutagenized with 0.6% ethyl methanesulfonate (EMS) dissolved in phosphate buffer for 8 h at room temperature. The mutagenized seeds were washed with sterilized water for 20 times, and were grown in soil for collection of M2 seeds.

### Generation of Mutants and Transgenic Plants

Mutations at 698th (-), 1120th (+) and 2216th (+) bp of coding sequence (CDS) of *ALA1* (Supplementary Figure 1B), and at the 951th (+) bp of CDS of *ALA2* (Supplementary Figure 5B) were introduced into the *rdr6* mutant through CRISPR/Cas9 method (Mao et al., 2013). The guide RNA of the CRISPR target was driven by U6 promoter, and Cas9 was under control of a CaMV35S promoter in a modified pCAMBIA1300 vector (Mao et al., 2013). Primers used for construction of vectors are listed in Supplementary Table 1. The constructs were introduced into *rdr6* mutants through agrobacterium-mediated flower dip method. The transgenic seeds were selected on MS containing 20 mg/L hygromycin, T2 plants were inoculated with CMV2aTΔ2b. Mutations of *ALA1* or *ALA2* were confirmed by sequencing.

The CDS of *ALA1* was cloned into the pCAMBIA1300 vector through SmaI and XbaI sites for fusion with three FLAG tags under the control of CaMV35S promoter, and introduced into the *ala1-2* using agrobacterium-mediated flower dip method.

### Whole-Genome Sequencing and Gene Cloning of *ENOR* Loci

The F2 population generated by crossing *enor1 rdr6* or *enor2 rdr6* with *rdr6* were inoculated with CMV2aTΔ2b. One hundred susceptible plants from F2 population were harvested at 21 days after inoculation to generate a bulked pool for DNA extraction with DNeasy Plant Maxi Kit (QIAGEN, Cat. 68163) and construction of DNA library. Whole genome sequencing was performed with the illumina HiSeq2000 platform. The softwares Skewer, Bowtie2 and SHOREmap were used to analyze the data and isolate mutations (Schneeberger et al., 2009; Sun and Schneeberger, 2015). The SNP-based Cleaved Amplified Polymorphic Sequences (CAPSs) markers generated from comparison of genome sequences of *enor1 rdr6* or *enor2 rdr6* with *rdr6* were used to assist mapping and cloning of *ENOR1* and *ENOR2*.

### Immunoblotting Analysis

The total proteins were extracted from plants at 21 days after inoculation with mock or CMV2aTΔ2b. Fifty microgram of total protein for each sample was quantified and loaded for detection of coat protein (CP) of CMV2aTΔ2b. The antibody against coat protein (anti-CP) of CMV2aTΔ2b was produced by Abmart company (Abmart^1^) with the recombinant protein of the 1st to 154th AA of CP. The anti-CP was used as first antibody (1:6000), and anti-rabbit immunoglobulin antibody was used as the secondary antibody respectively (1:3000). All of the experiments were repeated at least three biological times.

### GUS Staining

The L1 line transgenic for the β-glucuronidase (*GUS*) gene driven by 35S promoter (*35S::GUS*), in which the *GUS* activity is very low in all the expanded rosette leaves due to the post transcriptional gene silencing (Boutet et al., 2003), was crossed with *ala1-2* to generate *ala1-2* with L1 transgene (*35S::GUS*), named *ala1-2 35S::GUS*. Eighteen *ala1-2 35S::GUS* plants were used for histochemical staining of GUS using the method described previously (Shan et al., 2011).

### Quantitative Real-Time PCR Analysis

For **Figure 6A**, the expression of *ALA* family members was analyzed in Col-0 and *ala1-2* at 21 days after inoculation with mock or CMV2aTΔ2b. For Supplementary Figure 4, the accumulation of genomic RNA of CMV2aTΔ2b was analyzed in Col-0 and *ala1-2* at 21 days after CMV2aTΔ2b inoculation. The primers used for RNA detection of CMV2aTΔ2b were designed based on the conserved sequences from genomic RNA1 to RNA3 in the 3 prime end. The materials were harvested for RNA extraction using trizol (TRANSGENE, Cat.ET101-01), and reverse transcription was performed according to the kit (TRANSGENE, Cat. AT311-03). Quantitative real-time PCR was performed with EvaGreen 2^∗^qPCR MasterMix-Low ROX reagents (ABM, Cat. Mastermix-LR) using the ABI7500 real-time PCR system. *ACTIN8* was used as the internal control. All of the experiments were repeated at least three biological times. Primers used for quantitative real-time PCR analysis are listed in Supplementary Table 1.

### Phylogenetic Analysis

For the phylogenetic analysis shown in Supplementary Figure 6, the evolutionary history was inferred using the Neighbor-Joining method (Saitou and Nei, 1987). The optimal tree with the sum of branch length (4.45679805) is shown. The percentage of replicate trees in which the associated taxa clustered together in the bootstrap test (500 replicates) are shown next to the branches (Felsenstein, 1985). The evolutionary distances were computed using the Poisson correction method (Zuckerkandl and Pauling, 1965) and are in the units of the number of amino acid substitutions per site. The analysis involved all 12 amino acid sequences of ALA family. All positions containing gaps and missing data were eliminated. There were a total of 794 positions in the final dataset. Evolutionary analyses were conducted in MEGA6 (Tamura et al., 2013). The transcripts, including ALA1 (AT5G04930.1), ALA2 (AT5G44240.1), ALA3 (AT1G59820.1), ALA4 (AT1G17500.1), ALA5 (AT1G72700.1), ALA6 (AT1G54280.1), ALA7 (AT3G13900.1), ALA8 (AT3G27870.1), ALA9 (AT1G68710.1), ALA10 (AT3G25610.1), ALA11 (AT1G13210.1), and ALA12 (AT1G26130.2), were used for phylogenetic analysis.

### Subcellular Localization

Coding sequence of *ALA1* was cloned into the pJG054 vector for fusion with YFP under control of CaMV35S promoter (*YFP-ALA1*). The agrobacterium containing *YFP-ALA1* or the mCherry-ER-marker were resuspended in the infiltration buffer (10 mM MgCl_2_, 10 mM MES, 0.2 mM acetosyringone) for 3-5 h, and co-infiltrated into leaves of *N. benthamiana*. The fluorescence signals were collected with a Zeiss microscope (LSM710) at ∼50 h after co-infiltration. All of the experiments were repeated at least three biological times.

### Accession Numbers

The *Arabidopsis* Genome Initiative numbers for genes mentioned in this letter are as follows: ALA1 (AT5G04930), ALA2 (AT5G44240), ALA3 (AT1G59820), ALA4 (AT1G17500), ALA5 (AT1G72700), ALA6 (AT1G54280), ALA7 (AT3G13900), ALA8 (AT3G27870), ALA9 (AT1G68710), ALA10 (AT3G25610), ALA11 (AT1G13210), ALA12 (AT1G26130), RDR1 (AT1G14790), RDR6 (AT3G49500), and ACTIN8 (AT1G49240).

## Results

### Identification and Mapping of the *enor1* Mutant

**FIGURE 1:**
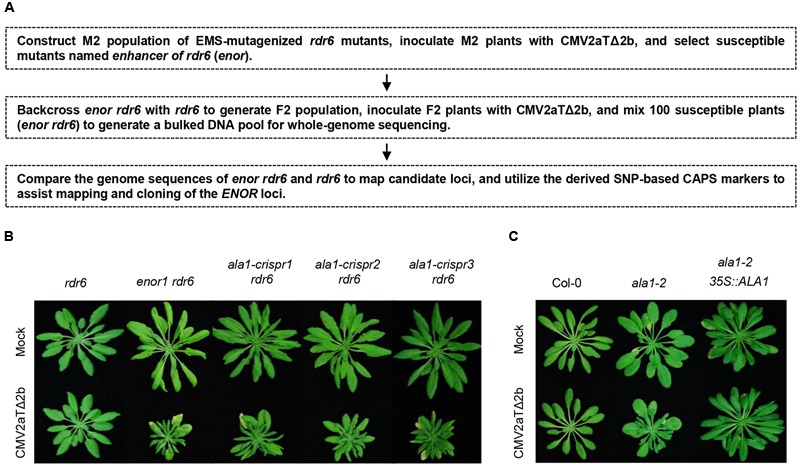
***ENOR1* encodes *ALA1* and mediates antiviral immunity. (A)** Schematic diagram of screening of *enor* (enhancer *of rdr6*) mutants and mapping of *ENOR* loci. **(B)** Phenotypes of *rdr6, enor1 rdr6* and the *ala1* mutants generated by CRISPR/Cas9 in *rdr6* (*ala1-crispr1, ala1-crispr2, ala1-crispr3*) at 21 days after infection with mock or CMV2aTΔ2b. **(C)** Phenotypes of Col-0, *ala1-2* and the *ala1-2* plant transgenic for the *ALA1* gene driven by 35S promoter (*ala1-2 35S::ALA1*) at 21 days after infection with mock or CMV2aTΔ2b.

We generated M2 population of EMS-mutagenized *rdr6*, and inoculated M2 with CMV2aTΔ2b to identify mutants that enhanced the susceptibility to CMV2aTΔ2b in *rdr6* (referred to as *enhancer of rdr6* [*enor*]), and utilized whole genome sequencing to assist mapping and cloning of *ENOR* loci (**Figure 1A**).

As shown in **Figure 1B**, the newly identified mutant *enor1* in the *rdr6* background, named *enor1 rdr6*, exhibited severely stunted and clustered leaves after infection with CMV2aTΔ2b. One fourth of F2 population from the cross between *enor1 rdr6* and *rdr6* were susceptible to CMV2aTΔ2b, demonstrating that *enor1* is a recessive mutation. In order to map the *ENOR1* locus, we generated a bulked pool of susceptible plants from the F2 population for whole-genome sequencing, screened mutations by comparing the sequences with SHOREmap methods (Schneeberger et al., 2009; Sun and Schneeberger, 2015), and mapped the *ENOR1* locus using CAPS markers (**Figure 1A**). We finally found that only a C to T mutation at the 2965th bp of CDS of AT5G04930, which causes a premature stop codon and generates a HaeIII-based CAPS marker, co-segregated with *enor1* (Supplementary Figures 1A,B).

### *ENOR1* Corresponds to *ALA1* and Is Essential for Antiviral Immunity

AT5G04930 encodes ALA1 (Lopez-Marques et al., 2014) that co-localizes with the mCherry-ER-marker (Supplementary Figure 2) (Lopez-Marques et al., 2012). To further genetically verify whether AT5G04930 (*ALA1*) corresponds to *ENOR1* and mediates antiviral immunity, we generated *ala1* mutants by the CRISPR/Cas9 genome editing method (Feng et al., 2014; Jia et al., 2016) in the *rdr6* background, and examined whether these *ala1-crispr rdr6* double mutants exhibit the viral symptoms similar to that of *enor1 rdr6* when inoculated with CMV2aTΔ2b. As shown in **Figure 1B**, all the *ala1-crispr rdr6* double mutants were severely susceptible to CMV2aTΔ2b, demonstrating that *ALA1* corresponds to *ENOR1* and is required for antiviral immunity.

We also obtained a T-DNA insertional mutant (Salk_056947, named *ala1-2*) of *ALA1* (Supplementary Figure 1B), and found that the *ala1-2* single mutant was mildly susceptible to CMV2aTΔ2b, less severe than *ala1-crispr rdr6* (**Figures 1B,C**), which also supports of the *ALA1* function in antiviral immunity. Moreover, we found that transgenic expression of *ALA1* under the control of CaMV 35S promoter fully restored the mutant phenotypes of *ala1-2* (**Figure 1C**).

### *ALA1* Acts Additively With *RDR1* and *RDR6* to Regulate Antiviral Immunity

**FIGURE 2:**
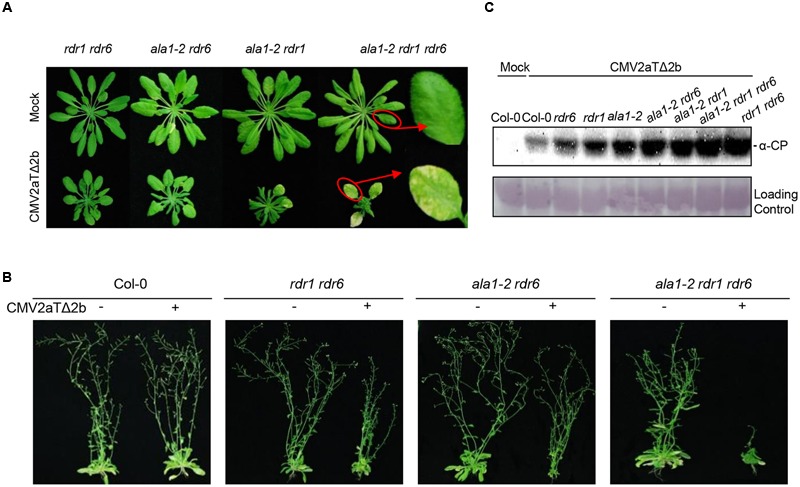
***ALA1* acts through both *RDR1*/*6*-related and -unrelated pathways to mediate antiviral immunity. (A)** Phenotypes of *rdr1 rdr6, ala1-2 rdr6, ala1-2 rdr1* and *ala1-2 rdr1 rdr6* at 21 days after infection with mock or CMV2aTΔ2b. **(B)** Phenotypes of Col-0, *rdr1 rdr6, ala1-2 rdr6* and *ala1-2 rdr1 rdr6* at 45 days after infection with mock or CMV2aTΔ2b. **(C)** Immunoblotting analysis to detect the accumulations of CMV2aTΔ2b from leaves of Col-0, *rdr6, rdr1, ala1-2, ala1-2 rdr6, ala1-2 rdr1, ala1-2 rdr1 rdr6* and *rdr1 rdr6* at 21 days after infection with mock or CMV2aTΔ2b. The coat protein (CP) of CMV2aTΔ2b was detected by anti-CP (α-CP) antibody. The large subunit of ribulose-1,5-bisphosphate was used as the loading control.

Further analyses of various double mutants and the triple mutant *ala1-2 rdr1 rdr6* showed that all the double mutants including *ala1-2 rdr6, ala1-2 rdr1, enor1 rdr6, ala1-crispr rdr6* and *rdr1 rdr6* exhibited similar symptoms after inoculation with CMV2aTΔ2b, which were much more severe than the single mutant *ala1-2*, while the triple mutant *ala1-2 rdr1 rdr6* showed the most severe symptoms with over-stunted newly born leaves and yellow old chlorotic leaves (**Figures 1B,C, 2A,B** and Supplementary Figure 3). These results suggest that *ALA1* functions additively with *RDR1* and *RDR6* to mediate plant immunity.

The immunoblot analysis with antibody against the CP of CMV2aTΔ2b showed that CMV2aTΔ2b accumulated much more in *ala1-2* than in wild-type, and that the double mutants (*ala1-2 rdr6, ala1-2 rdr1* and *rdr1 rdr6*) accumulated much more CP than the corresponding single mutants (**Figure 2C**). These results further demonstrate that *ALA1* acts additively with *RDR1* and *RDR6* to mediate RNAi-based antiviral immunity. Interestingly, the triple mutant *ala1-2 rdr1 rdr6* showed enhanced susceptibility compared with the double mutant *rdr1 rdr6* when inoculated with CMV2aTΔ2b, however, the accumulation of CMV2aTΔ2b was indistinguishable between the triple mutant *ala1-2 rdr1 rdr6* and the double mutant *rdr1 rdr6*, implying that *ALA1* mediates plant immunity through both a *RDR1/6*-related RNAi pathway and *RDR1/6*–unrelated pathways.

**FIGURE 3:**
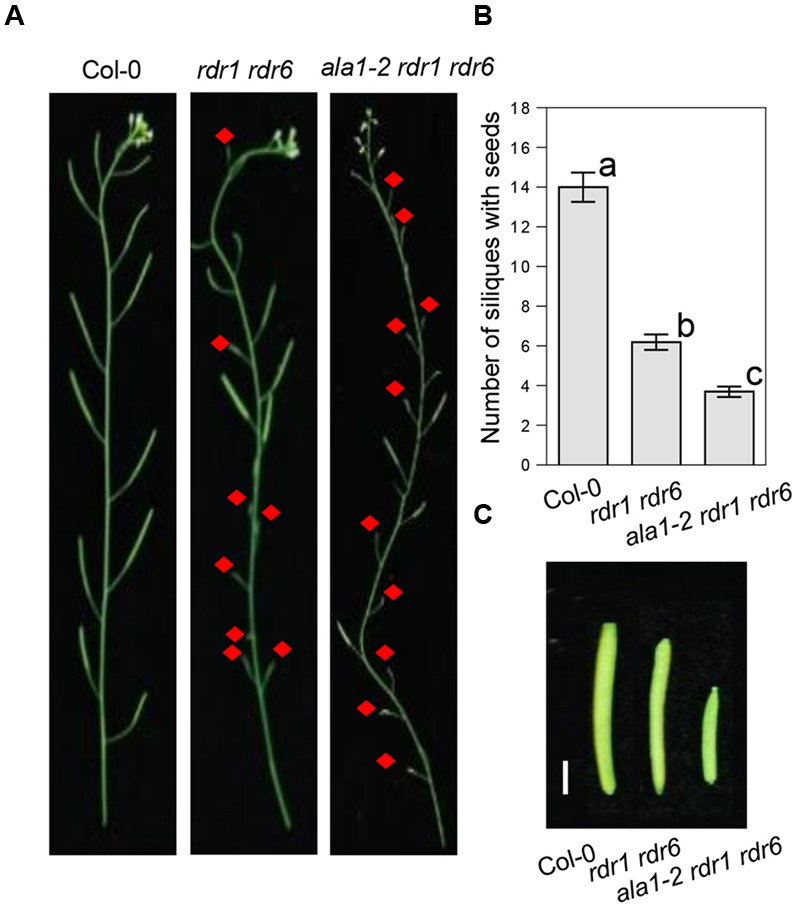
***ALA1* acts additively with *RDR1* and *RDR6* to regulate plant development. (A,B)** Phenotypes **(A)** and statistical analysis of numbers of siliques with seeds **(B)** of the main inflorescences from Col-0, *rdr1 rdr6* and *ala1-2 rdr1 rdr6*. Red arrows indicate sterile siliques. Errors represent ± SE. Lowercase letters indicate significant differences by one-way ANOVA analysis with SPSS software (*P* < 0.05). **(C)** Phenotypes of representative fertile siliques from Col-0, *rdr1 rdr6* and *ala1-2 rdr1 rdr6*. The bar represents 2 mm.

Further phenotypic analysis showed that the *ala1-2 rdr1 rdr6* triple mutant also displays developmental defects, including shorter siliques and less fertile siliques (**Figures 3A–C**). These results imply that *ALA1* may function additively with *RDR1* and *RDR6* to mediate RNAi-regulated plant development, consistent with the previous observations that RNAi, in addition to the RNAi-mediated plant immunity, also mediates plant developmental processes (Yoshikawa et al., 2005).

### *ALA1* Is Required for Gene Silencing

**FIGURE 4:**
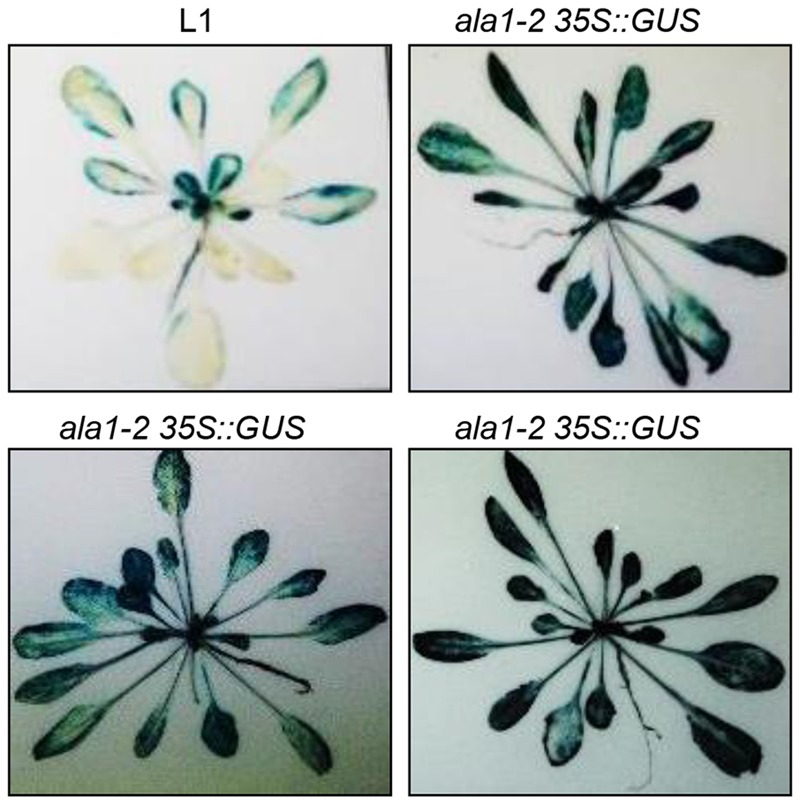
***ALA1* is involved in gene silencing.** The effect of *ala1-2* on post-transcriptional silencing of *35S::GUS* transgene of the L1 line. The *35S*::*GUS* transgene was post-transcriptionally silenced in the L1 line (Boutet et al., 2003); the homozygous *ala1-2* mutation prevents silencing of the *GUS* transgene in all expanded rosette leaves of the L1 line (*ala1 35S::GUS*), which was identified from F2 population of *ala1-2* and L1.

Having shown that *ALA1* acts additively with *RDR1* and *RDR6* in RNAi-based antiviral immunity and development, we further verified whether *ALA1* affects gene silencing via genetic cross of the *ala1-2* mutant with the L1, a transgenic silencing marker line where the *GUS* transgene driven by the CaMV35S promoter (*35S::GUS*) was silenced and expressed at low level (Boutet et al., 2003). As shown in **Figure 4**, the *GUS* activity was obviously increased in *ala1-2* (named *ala1-2 35S::GUS*). These data demonstrate that mutation in *ALA1* abolished the gene silencing on the *GUS* transgene driven by the 35S promoter, suggesting that *ALA1* is indeed required for gene silencing. Consistently, our quantitative real-time PCR analysis showed that the accumulation of CMV2aTΔ2b RNA in *ala1-2* was much higher than that in Col-0, further supporting the essential roles of *ALA1* in gene silencing and antiviral defense.

### *ALA2* Also Participates in Antiviral Immunity

**FIGURE 5:**
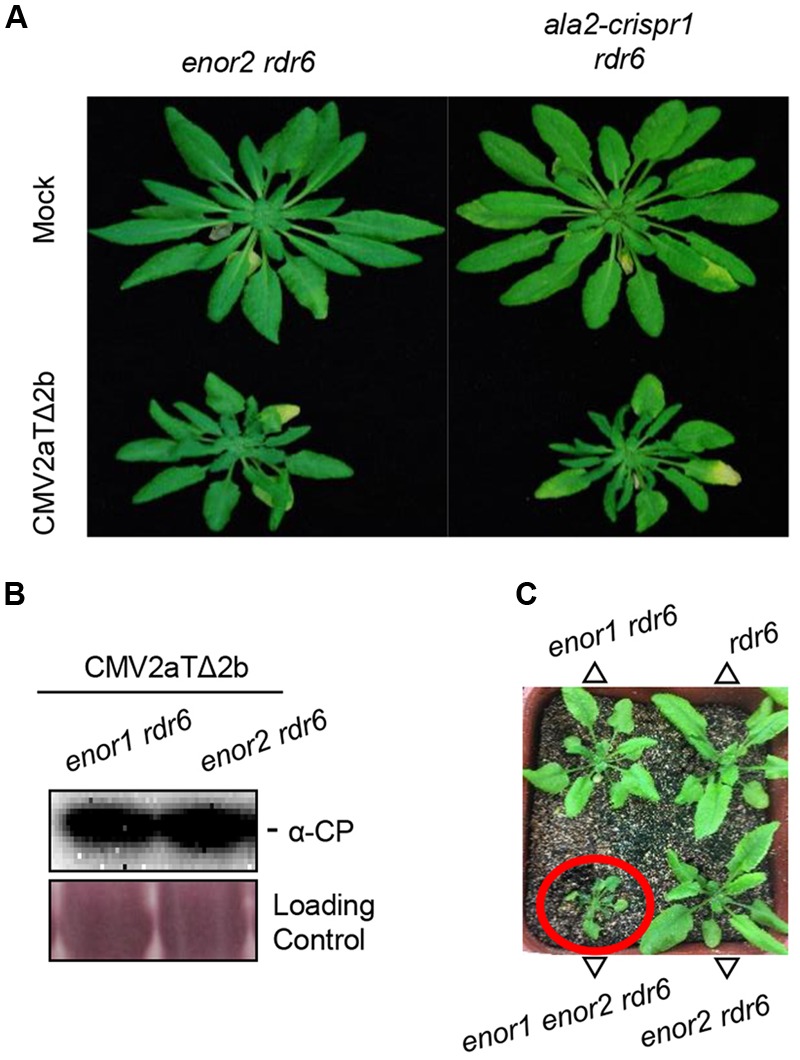
***ALA2* acts additively with *RDR6* and redundantly with *ALA1* in antiviral immunity. (A)** Phenotypes of *enor2 rdr6*, and the *ala2-crispr1* mutant generated by CRISPR/Cas9 in *rdr6* (*ala2-crispr1 rdr6*) at 21 days after infection with mock or CMV2aTΔ2b. **(B)** Immunoblotting analysis to detect the accumulations of CMV2aTΔ2b from leaves of *enor1 rdr6* and *enor2 rdr6* at 21 days after infection with mock or CMV2aTΔ2b. The coat protein (CP) of CMV2aTΔ2b was detected by anti-CP (α-CP) antibody. The large subunit of ribulose-1,5-bisphosphate was used as the loading control. **(C)** Phenotypes of 30-day-old seedlings of *rdr6, enor1 rdr6, enor2 rdr6*, and *enor1 enor2 rdr6*.

During the screening, we isolated a second enhancer mutant *enor2 rdr6* (**Figure 5A**), in which CP accumulation was similar with that in *enor1 rdr6* (**Figure 5B**). We further found that *ENOR2* encodes *ALA2* by performing the same mapping and identification procedures as *ENOR1* (Supplementary Figure 5A). The *ALA2* gene in *enor2 rdr6* contained a G to A mutation at the 1995th bp, leading to a premature stop codon (Supplementary Figure 5B), and mutation of *ALA2* by CRISPR/Cas9 in *rdr6* also resulted in severe susceptibility to CMV2aTΔ2b (**Figure 5A** and Supplementary Figure 5B), suggesting that *ALA2* mediates antiviral immunity. Moreover, we generated the *enor1 enor2 rdr6* triple mutant, and found that *enor1 enor2 rdr6* displayed severe developmental defects, including stunted leaves, which is similar with CMV2aTΔ2b-infected *enor1 rdr6* and *enor2 rdr6* (**Figure 5C**). This results (**Figure 5**) indicate that both *ALA1* and *ALA2* act additively with *RDR6* to mediate antiviral immunity and plant growth.

### Analysis of Other ALAs in Antiviral Immunity

**FIGURE 6:**
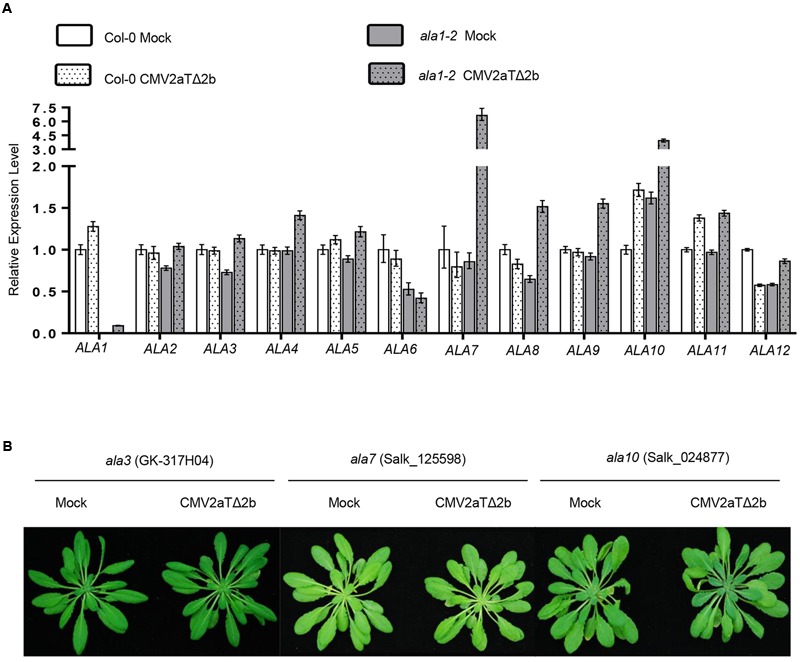
**Analysis of ALA members in antiviral silencing. (A)** Quantitative real-time PCR showed the relative expression levels of *ALA* family members in Col-0 and *ala1-2* after inoculation with mock or CMV2aTΔ2b. The data are means (±SE) from three biological repeats. **(B)** Phenotypes of the *ala3, ala7* and *ala10* mutants at 21 days after infection with mock or CMV2aTΔ2b.

Phylogenetic analysis of the ALA family proteins showed that ALA1 and ALA2 are the closest members, and other members are less related (Supplementary Figure 6). We observed that CMV2aTΔ2b infection dramatically induced the expression of *ALA7* and *ALA10* in *ala1-2*, but could not obviously affect the expression of other *ALAs* in wild-type and *ala1-2* (**Figure 6A**). We next investigated whether other ALA members play a role in antiviral immunity. The T-DNA insertion mutants of *ALA3* to *ALA12* were inoculated with CMV2aTΔ2b, and the results showed that none of these mutants were susceptible (**Figure 6B** and data not shown). It remains to be elucidated whether these ALAs function redundantly to mediate RNAi-based antiviral immunity and plant development.

## Discussion

It is well known that the RNAi pathway regulates plant growth, development and immunity. Previous studies have revealed that AGOs, DCLs, RDR1 and RDR6 are essential components of RNAi pathway (Ding and Voinnet, 2007; Qu et al., 2008; Wang et al., 2010; Cao et al., 2014). In this study, we developed an effective forward genetic screening using 2b-deficient CMV2aTΔ2b, and defined ALA1 and ALA2, membrane-localized proteins (**Figures 1, 5** and Supplementary Figure 2) (Lopez-Marques et al., 2010, 2012), as the new components in the RNAi pathway. ALA1 plays an essential role in gene silencing, and acts additively with RDR1/6 to mediate RNAi-based antiviral immunity and plant development (**Figures 2–4**). ALA2 also participates in antiviral defense and development, and acts redundantly with ALA1 in regulation of plant development in *rdr6* background (**Figure 5C**).

A recent study showed that AMP1, a novel key component in RNAi pathway, associates with AGO1 and mediates miRNA-targeted translational inhibition of mRNA on ER membrane (Li et al., 2013). miRNA-guided cleavage can also occur on ER membrane-bound polysomes (Li et al., 2016). These studies take ER into a central stage of small RNAs-mediated silencing (Ma et al., 2013; Li et al., 2016). On the other hand, viruses recruit ER membrane and manipulate lipid synthesis, transport and metabolism to form a circumstance essential for viral replication and morphogenesis (Fernández de Castro et al., 2016). Our finding that the ER membrane-localized ALA1 and ALA2 are essential players in silencing pathway and antiviral immunity would help to study and understand both the small RNAs machinery on ER membrane and the roles of lipid transport in silencing and antiviral defense. It would be interesting to investigate whether ALA1 and ALA2 associate with AMP1 and/or AGO1 to mediate gene silencing and antiviral immunity.

## Author Contributions

DX designed the study; BZ, HG, DW, and TQ performed experiments; DX, TQ, BZ, HG, GX, SS, HJ, and SZ analyzed the data. BZ, HG, TQ, and DX wrote the manuscript.

## Conflict of Interest Statement

The authors declare that the research was conducted in the absence of any commercial or financial relationships that could be construed as a potential conflict of interest.

## Footnotes

1 http://www.ab-mart.com.cn/

## References

[B1] Adenot X. Elmayan T. Lauressergues D. Boutet S. Bouche N. Gasciolli V. (2006). DRB4-dependent TAS3 trans-acting siRNAs control leaf morphology through AGO7. *Curr. Biol.* 16 927–932. 10.1016/j.cub.2006.03.035 16682354

[B2] Arribas-Hernandez L. Marchais A. Poulsen C. Haase B. Hauptmann J. Benes V. (2016). The slicer activity of ARGONAUTE1 is required specifically for the phasing, not production, of trans-acting short interfering RNAs in Arabidopsis. *Plant Cell* 28 1563–1580. 10.1105/tpc.16.00121 27354557 PMC4981131

[B3] Blevins T. Rajeswaran R. Shivaprasad P. V. Beknazariants D. Si-Ammour A. Park H. S. (2006). Four plant Dicers mediate viral small RNA biogenesis and DNA virus induced silencing. *Nucleic Acids Res.* 34 6233–6246. 10.1093/nar/gkl886 17090584 PMC1669714

[B4] Botella C. Sautron E. Boudiere L. Michaud M. Dubots E. Yamaryo-Botte Y. (2016). ALA10, a phospholipid flippase, controls FAD2/FAD3 desaturation of phosphatidylcholine in the ER and affects chloroplast lipid composition in *Arabidopsis thaliana*. *Plant Physiol.* 170 1300–1314. 10.1104/pp.15.01557 26620528 PMC4775126

[B5] Boutet S. Vazquez F. Liu J. Béclin C. Fagard M. Gratias A. (2003). Arabidopsis HEN1: a genetic link between endogenous miRNA controlling development and siRNA controlling transgene silencing and virus resistance. *Curr. Biol.* 13 843–848. 10.1016/S0960-9822(03)00293-8 12747833 PMC5137371

[B6] Cao M. Du P. Wang X. Yu Y. Q. Qiu Y. H. Li W. (2014). Virus infection triggers widespread silencing of host genes by a distinct class of endogenous siRNAs in *Arabidopsis*. *Proc. Natl. Acad. Sci. U.S.A.* 111 14613–14618. 10.1073/pnas.1407131111 25201959 PMC4209997

[B7] Diaz-Pendon J. A. Li F. Li W. X. Ding S. W. (2007). Suppression of antiviral silencing by cucumber mosaic virus 2b protein in *Arabidopsis* is associated with drastically reduced accumulation of three classes of viral small interfering RNAs. *Plant Cell* 19 2053–2063. 10.1105/tpc.106.047449 17586651 PMC1955711

[B8] Ding S. W. Li H. Lu R. Li F. Li W. X. (2004). RNA silencing: a conserved antiviral immunity of plants and animals. *Virus Res.* 102 109–115. 10.1016/j.virusres.2004.01.021 15068886

[B9] Ding S. W. Voinnet O. (2007). Antiviral immunity directed by small RNAs. *Cell* 130 413–426. 10.1016/j.cell.2007.07.039 17693253 PMC2703654

[B10] Felsenstein J. (1985). Confidence-limits on phylogenies - an approach using the Bootstrap. *Evolution* 39 783–791. 10.2307/2408678 28561359

[B11] Feng Z. Y. Mao Y. F. Xu N. F. Zhang B. T. Wei P. L. Yang D. L. (2014). Multigeneration analysis reveals the inheritance, specificity, and patterns of CRISPR/Cas-induced gene modifications in *Arabidopsis*. *Proc. Natl. Acad. Sci. U.S.A.* 111 4632–4637. 10.1073/pnas.1400822111 24550464 PMC3970504

[B12] Fernández de Castro I. Tenorio R. Risco C. (2016). Virus assembly factories in a lipid world. *Curr. Opin. Virol.* 18 20–26. 10.1016/j.coviro.2016.02.009 26985879

[B13] Garcia-Ruiz H. Takeda A. Chapman E. J. Sullivan C. M. Fahlgren N. Brempelis K. J. (2010). *Arabidopsis* RNA-dependent RNA polymerases and dicer-like proteins in antiviral defense and small interfering RNA biogenesis during Turnip Mosaic Virus infection. *Plant Cell* 22 481–496. 10.1105/tpc.109.073056 20190077 PMC2845422

[B14] Gomes E. Jakobsen M. K. Axelsen K. B. Geisler M. Palmgren M. G. (2000). Chilling tolerance in arabidopsis involves ALA1, a member of a new family of putative aminophospholipid translocases. *Plant Cell* 12 2441–2453. 10.1105/tpc.12.12.2441 11148289 PMC102229

[B15] Incarbone M. Dunoyer P. (2013). RNA silencing and its suppression: novel insights from in planta analyses. *Trends Plant Sci.* 18 382–392. 10.1016/j.tplants.2013.04.001 23684690

[B16] Jia Y. X. Ding Y. L. Shi Y. T. Zhang X. Y. Gong Z. Z. Yang S. H. (2016). The cbfs triple mutants reveal the essential functions of CBFs in cold acclimation and allow the definition of CBF regulons in *Arabidopsis*. *New Phytol.* 212 345–353. 10.1111/nph.14088 27353960

[B17] Li S. Le B. Ma X. Li S. You C. Yu Y. (2016). Biogenesis of phased siRNAs on membrane-bound polysomes in Arabidopsis. *Elife* 5:e22750 10.7554/eLife.22750 PMC5207768 27938667

[B18] Li S. Liu L. Zhuang X. Yu Y. Liu X. Cui X. (2013). MicroRNAs inhibit the translation of target mRNAs on the endoplasmic reticulum in *Arabidopsis*. *Cell* 153 562–574. 10.1016/j.cell.2013.04.005 23622241 PMC3694718

[B19] Lopez-Marques R. L. Poulsen L. R. Hanisch S. Meffert K. Buch-Pedersen M. J. Jakobsen M. K. (2010). Intracellular targeting signals and lipid specificity determinants of the ALA/ALIS P4-ATPase complex reside in the catalytic ALA alpha-subunit. *Mol. Biol. Cell* 21 791–801. 10.1091/mbc.E09-08-0656 20053675 PMC2828965

[B20] Lopez-Marques R. L. Poulsen L. R. Palmgren M. G. (2012). A putative plant aminophospholipid flippase, the Arabidopsis P4 ATPase ALA1, localizes to the plasma membrane following association with a beta-subunit. *PLoS ONE* 7:e33042 10.1371/journal.pone.0033042 PMC3326016 22514601

[B21] Lopez-Marques R. L. Theorin L. Palmgren M. G. Pomorski T. G. (2014). P4-ATPases: lipid flippases in cell membranes. *Pflugers. Arch.* 466 1227–1240. 10.1007/s00424-013-1363-4 24077738 PMC4062807

[B22] Ma X. Cao X. F. Mo B. X. Chen X. M. (2013). Trip to ER MicroRNA-mediated translational repression in plants. *RNA Biol.* 10 1586–1592. 10.4161/rna.26313 24100209 PMC3866237

[B23] Mao Y. Zhang H. Xu N. Zhang B. Gou F. Zhu J. K. (2013). Application of the CRISPR-Cas system for efficient genome engineering in plants. *Mol. Plant* 6 2008–2011. 10.1093/mp/sst121 23963532 PMC3916745

[B24] Martinez de Alba A. E. Elvira-Matelot E. Vaucheret H. (2013). Gene silencing in plants: a diversity of pathways. *Biochim. Biophys. Acta* 1829 1300–1308. 10.1016/j.bbagrm.2013.10.005 24185199

[B25] McDowell S. C. Lopez-Marques R. L. Cohen T. Brown E. Rosenberg A. Palmgren M. G. (2015). Loss of the *Arabidopsis thaliana* P4-ATPases ALA6 and ALA7 impairs pollen fitness and alters the pollen tube plasma membrane. *Front. Plant Sci.* 6:197 10.3389/fpls.2015.00197 PMC4404812 25954280

[B26] McDowell S. C. Lopez-Marques R. L. Poulsen L. R. Palmgren M. G. Harper J. F. (2013). Loss of the *Arabidopsis thaliana* P(4)-ATPase ALA3 reduces adaptability to temperature stresses and impairs vegetative, pollen, and ovule development. *PLoS ONE* 8:e62577 10.1371/journal.pone.0062577 PMC3646830 23667493

[B27] Morel J. B. (2002). Fertile hypomorphic ARGONAUTE (ago1) mutants impaired in post-transcriptional gene silencing and virus resistance. *Plant Cell Online* 14 629–639. 10.1105/tpc.010358 PMC150585 11910010

[B28] Parent J. S. Bouteiller N. Elmayan T. Vaucheret H. (2015). Respective contributions of Arabidopsis DCL2 and DCL4 to RNA silencing. *Plant J.* 81 223–232. 10.1111/tpj.12720 25376953

[B29] Poulsen L. R. Lopez-Marques R. L. Mcdowell S. C. Okkeri J. Licht D. Schulz A. (2008). The *Arabidopsis* P-4-ATPase ALA3 localizes to the Golgi and requires a beta-subunit to function in lipid translocation and secretory vesicle formation. *Plant Cell* 20 658–676. 10.1105/tpc.107.054767 18344284 PMC2329932

[B30] Qu F. Ye X. Morris T. J. (2008). *Arabidopsis* DRB4, AGO1, AGO7, and RDR6 participate in a DCL4-initiated antiviral RNA silencing pathway negatively regulated by DCL1. *Proc. Natl. Acad. Sci. U.S.A.* 105 14732–14737. 10.1073/pnas.0805760105 18799732 PMC2567185

[B31] Saitou N. Nei M. (1987). The Neighbor-Joining Method - a new method for reconstructing phylogenetic trees. *Mol. Biol. Evol.* 4 406–425. 3447015 10.1093/oxfordjournals.molbev.a040454

[B32] Schneeberger K. Ossowski S. Lanz C. Juul T. Petersen A. H. Nielsen K. L. (2009). SHOREmap: simultaneous mapping and mutation identification by deep sequencing. *Nat. Methods* 6 550–551. 10.1038/nmeth0809-550 19644454

[B33] Shan X. Wang J. Chua L. Jiang D. Peng W. Xie D. (2011). The role of Arabidopsis Rubisco activase in jasmonate-induced leaf senescence. *Plant Physiol.* 155 751–764. 10.1104/pp.110.166595 21173027 PMC3032464

[B34] Sun H. Schneeberger K. (2015). SHOREmap v3.0: fast and accurate identification of causal mutations from forward genetic screens. *Methods Mol. Biol.* 1284 381–395. 10.1007/978-1-4939-2444-8_19 25757783

[B35] Talmor-Neiman M. Stav R. Klipcan L. Buxdorf K. Baulcombe D. C. Arazi T. (2006). Identification of trans-acting siRNAs in moss and an RNA-dependent RNA polymerase required for their biogenesis. *Plant J.* 48 511–521. 10.1111/j.1365-313X.2006.02895.x 17076803

[B36] Tamura K. Stecher G. Peterson D. Filipski A. Kumar S. (2013). MEGA6: molecular evolutionary genetics analysis version 6.0. *Mol. Biol. Evol.* 30 2725–2729. 10.1093/molbev/mst197 24132122 PMC3840312

[B37] Wang X. B. Wu Q. Ito T. Cillo F. Li W. X. Chen X. (2010). RNAi-mediated viral immunity requires amplification of virus-derived siRNAs in *Arabidopsis thaliana*. *Proc. Natl. Acad. Sci. U.S.A.* 107 484–489. 10.1073/pnas.0904086107 19966292 PMC2806737

[B38] Xie Z. Fan B. Chen C. Chen Z. (2001). An important role of an inducible RNA-dependent RNA polymerase in plant antiviral defense. *Proc. Natl. Acad. Sci. U.S.A.* 98 6516–6521. 10.1073/pnas.111440998 11353867 PMC33500

[B39] Yoshikawa M. Peragine A. Park M. Y. Poethig R. S. (2005). A pathway for the biogenesis of trans-acting siRNAs in *Arabidopsis*. *Genes Dev.* 19 2164–2175. 10.1101/gad.1352605 16131612 PMC1221887

[B40] Zhang X. Yuan Y. R. Pei Y. Lin S. S. Tuschl T. Patel D. J. (2006). Cucumber mosaic virus-encoded 2b suppressor inhibits *Arabidopsis* Argonaute1 cleavage activity to counter plant defense. *Genes Dev.* 20 3255–3268. 10.1101/gad.1495506 17158744 PMC1686603

[B41] Zuckerkandl E. Pauling L. (1965). Molecules as documents of evolutionary history. *J. Theor. Biol.* 8 357–366. 10.1016/0022-5193(65)90083-4 5876245

