# Supplementary material for: Arabidopsis ALA1 and ALA2 Mediate RNAi-Based Antiviral Immunity
Source: Front Plant Sci. 2017 Apr 7;8:422. doi: 10.3389/fpls.2017.00422 (PMC5383662; doi:10.3389/fpls.2017.00422)
Supplement: Supplementary file 1 [file Presentation_1.pdf]

Supplementary Figures

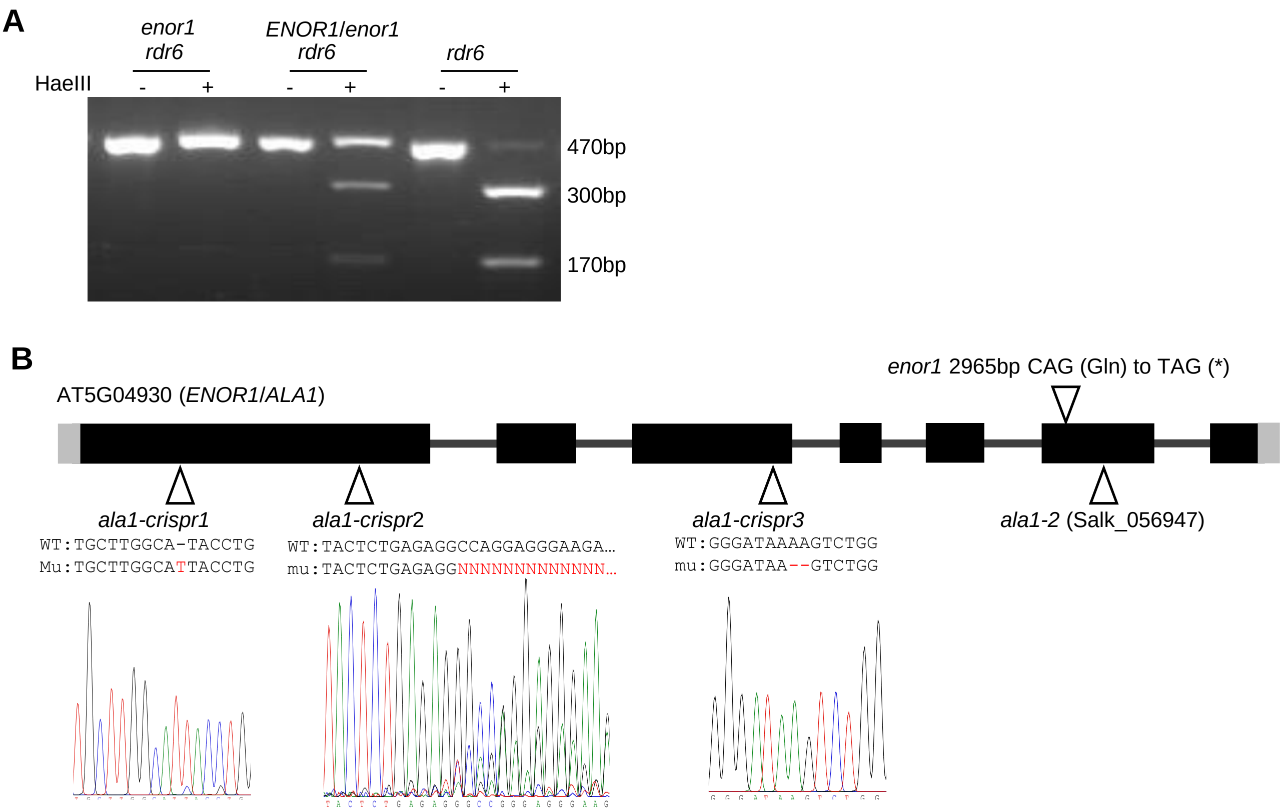

Figure S1. Molecular identification of *ENOR1*.

(A) The nucleotide mutation in *ENOR1* (*ALA1*) of *enor1 rdr6* led to a mutation (GGCC to GGCT) of the recognition sequence of HaeIII. The 470bp PCR product spanning the HaeIII site can be cleaved into fragments of 170bp and 300bp in *rdr6* but not in *enor1 rdr6*.

(B) Schematic diagram shows the structure of *ENOR1*/*ALA1* (AT5G04930) and the mutations of *ala1* mutants. The positions of mutation sites of *ALA1* in *enor1 rdr6*, *ala1-2* (Salk_056947) and *ala1* mutants (*ala1-crispr1*, *ala1-crispr2*, and *ala1-crispr3*) generated by CRISPR/Cas9 are indicated by open triangles. The *ALA1* gene in *enor1 rdr6* contains a C-to-T mutation at 2965th bp of CDS, converting a Gln codon (CAG) to a premature stop codon (TAG). The *ALA1* gene in *ala1-crispr1* contains a 1-bp insertion, and that in *ala1-crispr3* has a 2-bp deletion. The *ALA* gene in *ala1-crispr2* contains chimeric mutations represented with "NNN..." from the 1120th bp of ALA1 CDS.

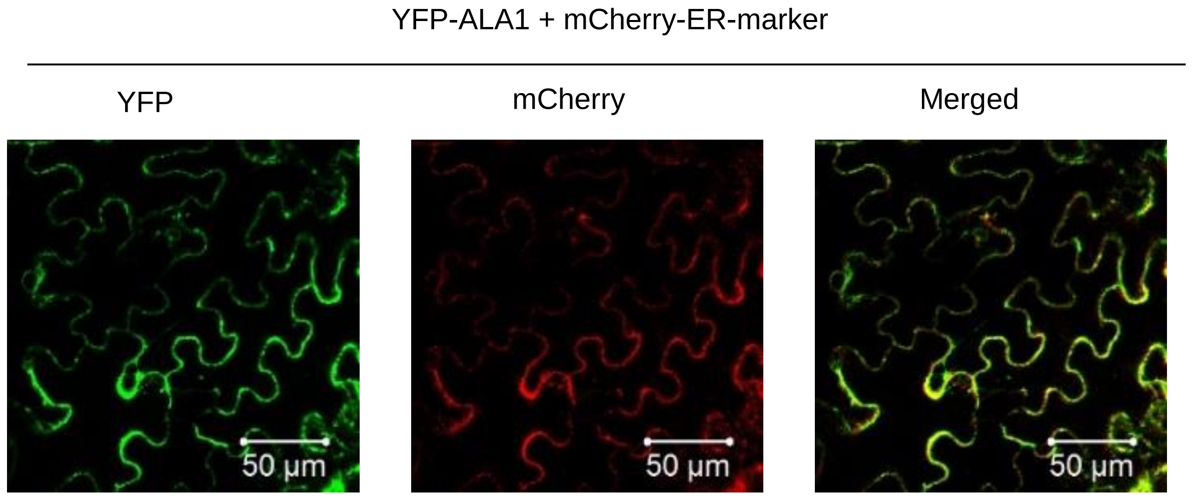

Figure S2. Subcellular localization of ALA1.

ALA1 with fusion of YFP (YFP-ALA1) was co-infiltrated with the mCherry-ER marker in leaves of *Nicotinana benthamiana*, and the fluorescence signals were detected 50h after co-infiltration.

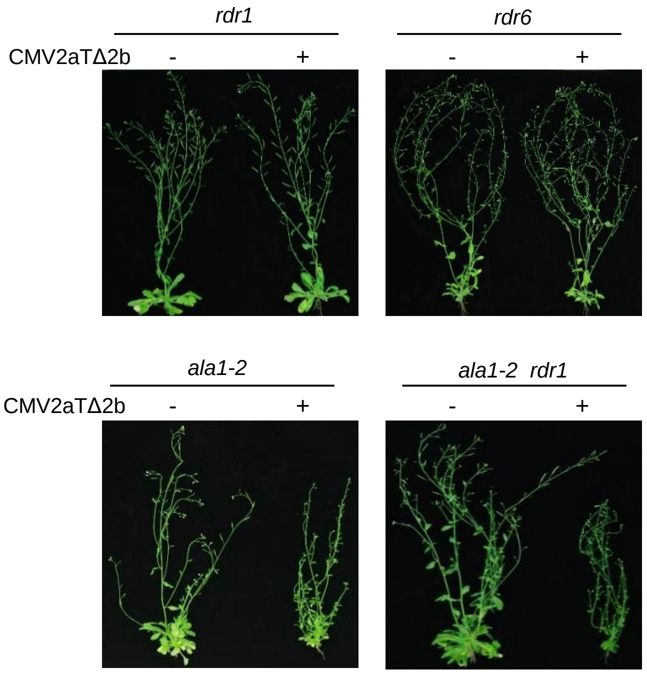

Figure S3. Pathogenic responses of *rdr1, rdr6, ala1-2* and *ala1-2 rdr1*.

The plants were photographed at 45 d after infection with mock or CMV2aTΔ2b.

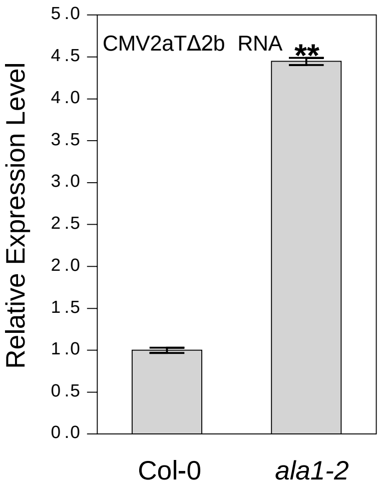

Figure S4. Accumulation of CMV2aTΔ2b genomic RNA in inoculated Col-0 and *ala1-2*. Quantitative real-time PCR analysis of the accumulation of CMV2aTΔ2b RNA in Col-0 and *ala1-2* at 21 d after inoculation. The data are means (±SE) from three biological repeats. The asterisks represent significant differences in mutants compared with Col-0 by Student's t-test, **P< 0.01.

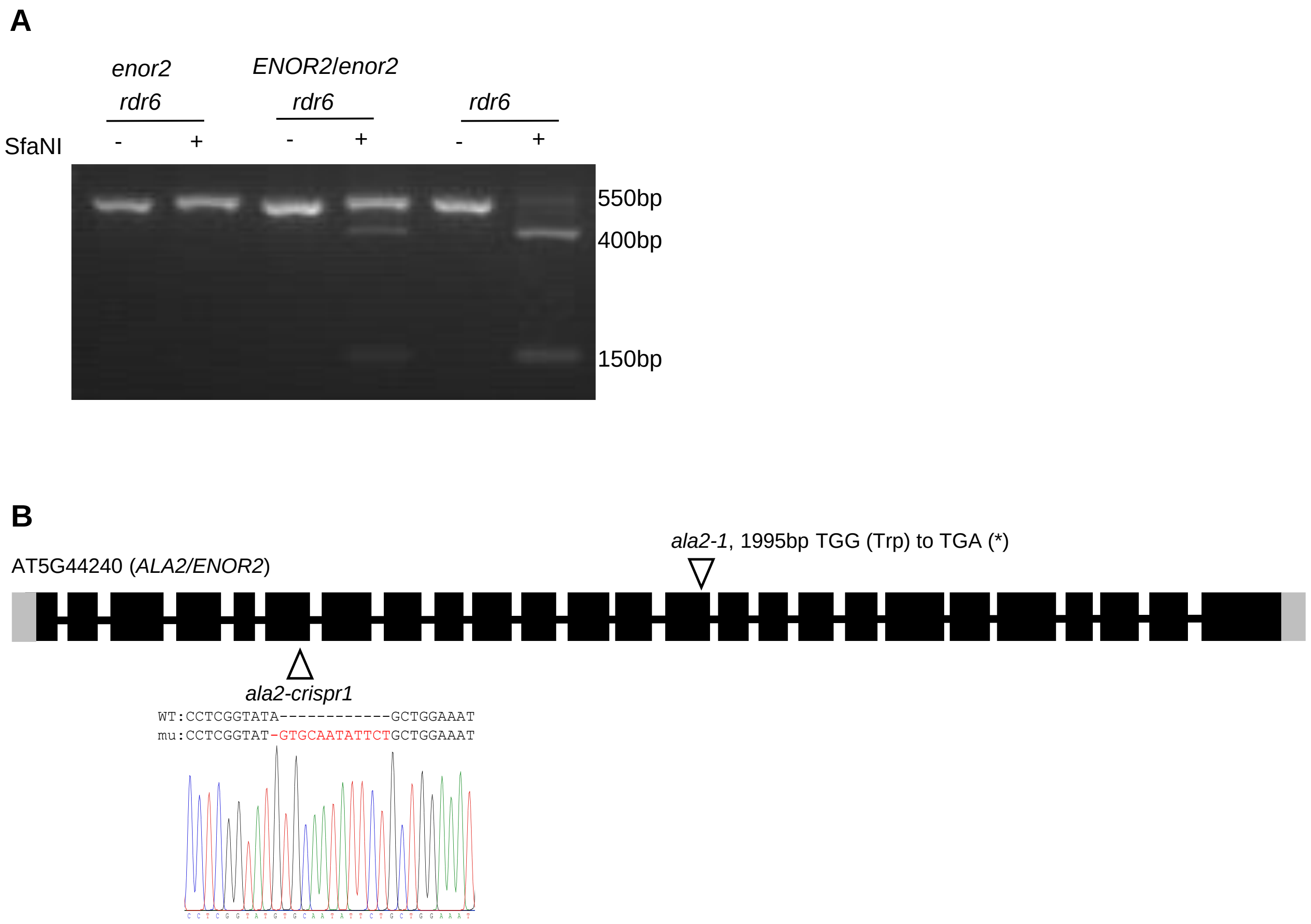

Figure S5. Molecular identification of *ENOR2*.

(A) The nucleotide mutation in the *ENOR2* (*ALA2*) gene of *enor2 rdr6* led to a mutation (GATGC to AATGC) of the recognition sequence of SfaNI. The 550bp PCR product spanning the SfaNI site can be cleaved into fragments of 150bp and 400bp in *rdr6* but not in *enor2 rdr6*.

(B) Schematic diagram shows the structure of *ENOR2*/*ALA2* (AT5G44240) and the mutations of *ala2* mutants. The positions of mutation sites of *ALA2* are indicated by open triangles. The *ALA2* gene in *enor2 rdr6* contains a G-to-A mutation at 1995th bp of CDS, converting a Trp codon (TGG) to a premature stop codon (TGA). The *ALA2* gene in *ala2-crispr1* generated by CRISPR/Cas9 contains a 1-bp deletion and a 12-bp insertion.

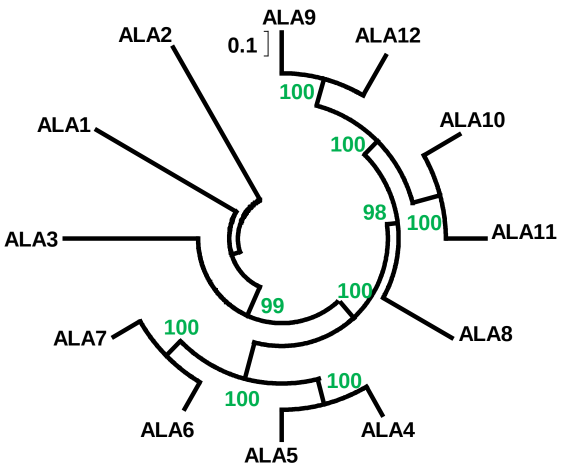

Figure S6. Phylogenetic analysis of Arabidopsis P4-type ATPase.

0.1 stands for distance scale. The numbers in green represent node statistics.

Supplementary Tables

Table S1. List of primers used in this work.

rdr1-1-TDNA	Forward	TGTCACCAGAAGAAGCTCCAG
rdr1-1-TDNA	Reverse	TTCCTTTCGTATAGTTGGGCC
rdr6-15-TDNA	Forward	TCTCCGCATTGCTTCTATTTC
rdr6-15-TDNA	Reverse	GCTGCAAAATAAGCACAAAGC
Salk_056947	Forward	GCCATTGGTGATGGTAATGAC
Salk_056947	Reverse	CCCTGTTTAAGAAAAAGCGATG
GK317H04-TDNA	Forward	TTTCTGATCGTGTCATTATTCAACTG
GK317H04-TDNA	Reverse	TGTTGTAGAAATCGAATTACCCTG
Salk_125598-TDNA	Forward	GGGGCGTCGTAGAATAAGATC
Salk_125598-TDNA	Reverse	GCTGATGAAGGAGACAAGCAC
Salk_024877-TDNA	Forward	GTACGGAGCTGTCGTTTTCAC
Salk_024877-TDNA	Reverse	AGAACTCGAACCCGAACTCTC
ala1-cripsr1-sg	Forward	GGTTTCCTGCTTGGCATACCGTTTTAGAGCTAGAAATAGC
ala1-cripsr1-sg	Reverse	GGTATGCCAAGCAGGAAACCAATCACTACTTCGACTCTAG

ala1-cripsr1-sg-cas9	Forward-HindIII	CCCTTAAGCTTCGTTGAACAACGGAAAC
ala1-cripsr1-sg-cas9	Reverse-SalI	TATCAAGCTTATCGATACCGTCGAC
ala1-cripsr1-seq	Forward	ACGGTTTGAGTTCACTGGGA
ala1-cripsr1-seq	Reverse	TGGCCTCTCAGAGTAGTCCT
ala1-cripsr2-sg	Forward	GAGGACTACTCTGAGAGGCCGTTTTAGAGCTAGAAATAGC
ala1-cripsr2-sg	Reverse	GGCCTCTCAGAGTAGTCCTCAATCACTACTTCGACTCTAG
ala1-cripsr2-sg-cas9	Forward-HindIII	CCCTTAAGCTTCGTTGAACAACGGAAAC
ala1-cripsr2-sg-cas9	Reverse-SalI	TATCAAGCTTATCGATACCGTCGAC
ala1-cripsr2-seq	Forward	GGGCTTTAGGGGTTGTTGTG
ala1-cripsr2-seq	Reverse	CTTCTGTTGCCTTGCCAGTT
ala1-cripsr3-sg	Forward	GGATTGCAGGGATAAAAGTCGTTTTAGAGCTAGAAATAGC
ala1-cripsr3-sg	Reverse	GACTTTTATCCCTGCAATCCAATCACTACTTCGACTCTAG
ala1-cripsr3-sg-cas9	Forward-HindIII	CCCTTAAGCTTCGTTGAACAACGGAAAC

ala1-cripsr3-sg-cas9	Reverse-SalI	TATCAAGCTTATCGATACCGTCGAC
ala1-cripsr3-seq	Forward	TACGGTGGCGTCATACATGA
ala1-cripsr3-seq	Reverse	ATCCCTACCCCAACATCAGC
ala2-cripsr-1-sg	Forward	GTTCTGGTCCTCGGTATAGCGTTTTAGAGCTAGAAATAGC
ala2-cripsr-1-sg	Reverse	GCTATACCGAGGACCAGAACAATCACTACTTCGACTCTAG
ala2-cripsr-1-sg-cas9	Forward-HindIII	CCCTTAAGCTTCGTTGAACAACGGAAAC
ala2-cripsr-1-sg-cas9	Reverse-SalI	TATCAAGCTTATCGATACCGTCGAC
ala2-cripsr1-seq	Forward	GCAAACATGCGCTTATTTCCG
ala2-cripsr1-seq	Reverse	GTCTGCCTCTGTCCTGAACT
enor1-caps	Forward	GGACACTGGAATTACCAAAGGATG
enor1-caps	Reverse	GTGAAGATTAACCACCACAACTGC
enor2-dcaps	Forward	ATTTAAATGTCCAGGACTAGTGAG
enor2-dcaps	Reverse	TCAGAGCAATTTCTAGAGC
ALA1-35SOE-3FLAG	Forward-SmaI	GGGGGGCCCGGGATGGATCCCAGGAAATCAATTG
ALA1-35SOE-3FLAG	Reverse-XbaI	GGGGGGTCTAGATCATCTCCGTGGAGGATCCTGAATC

ALA1-PJG054-YFP	Forward	CGACGACAAGACCGTGGATCCCAGGAAATCAATTGA
ALA1-PJG054-YFP	Reverse	GAGGAGAAGAGCCGTCATCATCTCCGTGGAGGATCCTGAATC
ALA1-qRT	Forward	ATGTTATACCCACACTCCCTGGT
ALA1-qRT	Reverse	ACATCCATGTCTTGCCCACT
ALA2-qRT	Forward	ACTTGTGGGAGCAATTCAGC
ALA2-qRT	Reverse	CACAGGGGTGATAAGTGACCA
ALA3-qRT	Forward	AGGCACTTGAAAGGACATGG
ALA3-qRT	Reverse	TGTTTGGTTGCTCACACTGAA
ALA4-qRT	Forward	GCCCTTGGAGTCTTTGAACA
ALA4-qRT	Reverse	TGTTGGTACAACGCTGGAAA
ALA5-qRT	Forward	ATGTTTCTTCCGAGATCTGCTT
ALA5-qRT	Reverse	GGTTCTTTGTGCCTTGTTGG
ALA6-qRT	Forward	GTAAAGGCGCTGACAGCAT
ALA6-qRT	Reverse	CCCGCTTCACCATATACATTC
ALA7-qRT	Forward	AGAAAGGGGTGCCTCAATG
ALA7-qRT	Reverse	ACCCTGTCTCAGCAAACTGC
ALA8-qRT	Forward	AACCGGTTGTTGTTGCTTTC
ALA8-qRT	Reverse	TCCATGTTTCGCAAGTCTTTT
ALA9-qRT	Forward	GTGCGTCTGTCATTTGTTGC
ALA9-qRT	Reverse	TCCAGATTTTACTAGTCTTGTCACCA

ALA10-qRT	Forward	GGGAAGCTCTTGCTGTTGTC
ALA10-qRT	Reverse	ACGACCGTTCTTTGCAAGTC
ALA11-qRT	Forward	CGACGAAGATGGGAGACTTC
ALA11-qRT	Reverse	CCGTTTTTGGCTAGTCTTTCA
ALA12-qRT	Forward	AAAGATTTGGGTTTTGACTGGA
ALA12-qRT	Reverse	TTCATGTCTCGTCTGAGCAAA
ACTIN8-qRT	Forward	TCAGCACTTTCCAGCAGATG
ACTIN8-qRT	Reverse	CTGTGGACAATGCCTGGAC
CMV-RNA-qRT	Forward	GTGAACGGGTTGTCCATCCAGC
CMV-RNA-qRT	Reverse	TGAAACTAGCACGTTGTGCTAG
